# Bionanocomposites based on chitosan intercalation in designed swelling high-charged micas

**DOI:** 10.1038/s41598-019-46495-z

**Published:** 2019-07-16

**Authors:** María D. Alba, Agustín Cota, Francisco J. Osuna, Esperanza Pavón, Ana C. Perdigón, Florian Raffin

**Affiliations:** 1 Instituto Ciencia de los Materiales de Sevilla, ICMS, (CSIC-US), Avda. Americo Vespucio, 49, 41092 Sevilla, Spain; 2 Laboratorio de Rayos X, CITIUS, (Universidad de Sevilla), Avda. Reina Mercedes, 4, 41012 Sevilla, Spain; 3 Departamento de Química e Ingeniería de Procesos y Recursos, Universidad Cantabria. Avda. Los Castros s/n, 39005 Santander, Spain; 4 École Nationale Supérieure de Chimie de Lille (E.N.S.C.L). Cité Scientifique – Bât 7. Avenue Mendeleïev CS 90108, 59652 Villeneuve D’ascq, Cedex, France

**Keywords:** Chemistry, Materials science

## Abstract

Bionanocomposites based on layered inorganic components, as clays, and polymers of biological origin, as chitosan, have a major impact in medical and environmental fields, being economical and environmentally friendly materials. Na-M*n* micas (*n* = 2 and 4) with controlled surface charge, high cation exchange capacity and swelling behaviour, are attractive inorganic composite components that exhibit improved adsorption properties compared to other inorganic solids which makes them potentially useful for bionanocomposites. The goal of this research was to explore the potential use of those synthetic brittle micas to form eco-friendly bionanocomposites with chitosan biopolymer. Hence, chitosan-mica bionanocomposites were prepared by ion-exchange reaction between chitosan solution and synthetic high charge mica. X-ray diffraction, Fourier transform infrared spectroscopy, thermal analysis, MAS-NMR spectroscopy and zeta-potential have been employed for bionanocomposites characterization. The results showed that the adsorption of chitosan is effective, although a chitosan portion remains in the outer surface being hydrogen-bonded to the tetrahedral sheet of the silicate.

## Introduction

Bionanocomposites are nanocomposites composed of a biopolymer and an inorganic solid^1^. Polymers of biological origin are the primary constituents for bionanocomposite preparation, among them chitosan, a natural polysaccharide, is being increasingly applied^2–5^. Chitosan is the second most abundant organic compound on Earth and is a deacetylate derivative of insects, molluscs, crustaceans, and fungi chitin^2^. The biopolymer chitosan offers excellent biological and physicochemical characteristics^6,7^, their derivate composites are easy to prepare^8^ and have been developed to adsorb heavy metals and dyes from wastewater^9–12^, with better behaviour than conventional adsorbents^8^.

Nanoclays are the most popular inorganic components in the preparation of bionanocomposites due to their low cost, high availability, high cation exchange capacity, strong adsorption ability and high surface area^13–16^. Montmorillonite^12,17–26^, laponite^27–29^, hectorite^30,31^, and saponite^32,33^ have been extensively studied in the literature. The introduction of chitosan in their structures leads to an improvement in their natural adsorption capabilities, forming biodegradable, economical and environmentally friendly materials^34^. Thus, chitosan-clay nanocomposites as bio-adsorbents have been successfully reported for the removal of water pollutants such dyes and heavy metals (tungsten, As(V) and Cr(VI))^9,12^.

Controlling the biopolymer/nanoclay interactions can open new route to generate smart nanomaterials with interesting technological properties, such as reliable thermal stability, hydrophobicity surface, and relevant environmental applications. In fact, many authors^35,36^ have expressed the need to explore parameters such as time, initial chitosan concentration, chitosan/clay ratio and type of the clay to optimize the chitosan loading. Among them, the nature of the clay, 2:1 phyllosilicate, is one of the critical parameter^35^ since electrostatic attractive forces improve the controlled drug release of those bionanocomposites^37–39^. Therefore, design synthetic nanoclays, that allow controlling their structural composition, can be a priceless help to obtain a deep knowledge of the bioloymer/nanoclay interaction.

Synthetic swelling high-charged fluorophlogopites, Na-M*n* (*n* = 2 and 4 represents the layer charge), are attractive nanoclays adsorbents for their unique combination of high cation exchange capacity (CEC, 247 meq/100 g of clay for Na-M2 and 468 meq/100 g of clay for Na-M4), swelling behaviour and high layer charge. Moreover, their physical and chemical properties can be easily tuneable by an appropriate synthesis method^40–43^. These synthetic clay minerals have been successfully applied for the selective removal of heavy metals^40^, highly radioactive ions^44,45^ and hydrocarbon molecules after an appropriate surface modification^46,47^. In fact, they overcome the limited CEC and adsorption capacity of natural nanoclays, and, in particular, organo-functionalized swelling high charged micas have already shown their excellent sorption capacity for the non-ionic organic pollutants benzene, toluene and phenol^47^. Thus, they could be used as inorganic former of bionanocomposites obtaining better bioadsorbents.

Up to our knowledge, bionanocomposites formed with the combination of swelling brittle micas, Na-M*n*, and chitosan biopolymer has never been explored although their joint properties and controlled synthesis will make this new bionanocomposite opens new routes in the field. Thus, the objective of this research was to evaluate the potential use of those synthetic mica, that overcome the limited CEC and adsorption capacity of the natural clay minerals, as inorganic component of an eco-friendly bionanocomposite. Even more, several synthesis recipes have been explored (Table S1) for the optimization of the chitosan loading. The bionanocomposites were characterized by Thermal analysis (TG), X-ray diffraction (XRD), Fourier Transform Infrared spectroscopy (FTIR), Zeta-potential and Solid State Nuclear Magnetic Resonance spectroscopy (MAS-NMR).

## Results and Discussion

The stability and adsorbed amount of chitosan was evaluated with thermogravimetrical analysis. The DTG plots (Fig. S2) show mainly two weight loss regions: the first one in the range 25–200 °C, related to the adsorbed water molecules, and the second one, between 200 and 900 °C, due to dehydration, polymer chains depolymerisation and decomposition.

The weight loss between room temperature and 200 °C is 7.6% and 6.8% in the starting silicates, Na-M2 and Na-M4, respectively. The bionanocomposites show higher losses than the starting inorganic mica, up to 10.6% (Table 1) due to the high water-retention capacity of chitosan.

**Table 1:** Water content, chitosan content and zeta-potential of Ch-NaM*n* (*n*=2 and 4).

Mica-*n*	Mica-2			Mica-4		
	H_2_O g/100g	Ch g/100g	Zeta potential (mV)	H_2_O g/100g	Ch g/100g	Zeta potential (mV)
Na-M*n*	7.6	—	−34.5 ± 0.9	6.8	—	−32.2 ± 0.8
Ch	8.7	98.6	+16.7 ± 1.5	8.7	98.6	+16.7 ± 1.5
Ch-NaM*n*-A	7.8	1.0	−0.8 ± 0.1	6.9	3.9	+5.5 ± 0.3
Ch-NaM*n*-B	8.5	1.4	+15.9 ± 1.2	7.1	3.9	+1.5 ± 0.9
Ch-NaM*n*-C	8.2	2.4	+5.7 ± 0.1	7.8	6.0	−2.4 ± 0.2
Ch-NaM*n*-D	8.3	10.3	+1.9 ± 1.1	10.5	7.9	+1.3 ± 0.9
Ch-NaM*n*-E	10.3	8.3	+23.2 ± 0.1	10.6	10.3	+24.4 ± 0.6

The adsorbed chitosan amount is calculated from the total weight loss in the 200 °C-900 °C temperature range. It is referenced to the dried sample and corrected taken into account that in this temperature range: (i) Na-M*n* samples loss 2.5 and 1.5% of weigh (for *n* = 2 and 4, respectively) due to dehydroxylation, and, (ii) 98.6% of the chitosan sample decomposes (Table 1). The obtained values are comprised between 1 and 10.3 g of chitosan per 100 g of mica. Mica layer charge influences the amount of adsorbed chitosan, being higher in Mica-4 than in Mica-2, in good agreement with the higher cation exchange capacity, CEC, of Mica-4 (468 meq/100 g) *vs* Mica-2 (247 meq/100 g).

There is no influence of acetic acid concentration (Ch-NaM*n*-A and Ch-NaM*n*-B) in the adsorption of chitosan. Chitosan is a weak polyelectrolyte and their amine groups are protonated under acidic conditions, with pKa ~ 6.5^48^, thus, the pH of the solution during the chitosan loading experiment was fixed at 4.9, to ensure that the amine groups are fully protonated.

However, the initial concentration of chitosan does have an influence (Ch-NaM*n*C vs Ch-NaM*n*-D). Yu *et al*.^36^ observed a linear increasing of loaded chitosan with initial chitosan concentration up to a plateau is reached. In Mica-2, the adsorption notably increases when the initial concentration of chitosan is raised (Ch-NaM2-C and Ch-NaM2-D), as it was previously reported for montmorillonite^20^. However, this increment is not observed in Mica-4 (Ch-NaM4-C and Ch-NaM4-D) due to the partial compensation of the layer charge by protons in the acid reaction media^45,49^.

Chitosan adsorption was enhanced for the highest layer charge nanoclay, except for Ch-NaM*n*-D samples. Na-M2 and Na-M4 differ in the ratio of isomorphous substitution of aluminium by silicon in the tetrahedral sheet, and, hence, in the number of negative charges per unit cell in the aluminosilicate layer. As the layer charge increases from 2 to 4, two opposite forces control the adsorption: the electrostatic interactions between the interlayer cations and the framework difficult the exchange mechanism whereas the high cation exchange capacity allows that more molecules can be hosted in the interlayer space. The adsorption of chitosan was lower in Ch-NaM4-D than in Ch-NaM2-D, as previously observed by Osuna *et al*.^42^ for the adsorption of Cs^+^ at severe conditions due to a higher layer charge compensation by hydronium in Mica-4.

Replacing water by acetic acid in the dissolving process improved Na-M4 chitosan adsorbent ability whereas the opposite occurred in Na-M2 sample (Ch-NaM*n*-D and Ch-NaM*n*-E). Günister *et al*.^50^ observed that the addition of acetic acid to the aqueous dispersions increases the interlayer space of clay minerals, facilitating chitosan adsorption. As observed in our samples, this effect is more evident when layer charge increases.

The DTG curves in the range between 200 °C to 900 °C (Fig. S2) illustrate the thermal decomposition behaviour of chitosan. Ch-NaM*n* bionanocomposites exhibit higher thermal decomposition temperature than pure chitosan, shifting ca. 165 °C, due to the intercalation of chitosan into the mica that stabilizes the organic matter^51,52^. Bionanocomposites exhibit two peaks on the DTG curves, one at lower temperature, similar to pure chitosan and other at higher temperature due to chitosan-mica nanocomposites. This suggest that a certain amount of polymer molecules is outside adhered to other chitosan molecules by Van der Waals forces due to the difficulty to penetrate into the interlayer space in high charged micas^53^.

Nanoclays surface is affected by chitosan incorporation in the framework, as it can be observed in the zeta-potential values (Table 1) obtained for chitosan-mica nanocomposites. Zeta-potential of mica particles is negative, as expected, −34.5 mV in Na-M2 and −32.2 mV in Na-M4, whereas a positive value, +16.7 mV, is observed for pure chitosan. When bionanocomposites are formed, NaM*n* zeta-potential is turned positive, indicating that mica particles are covered by ionized chitosan molecules^20,53^, this effect is quite noticeable for Ch-NaM*n*-E, where acetic acid is used as solvent for the mica dispersions. In those samples, the high zeta-potential values observed (+23.2 and +24.4 for Ch-NaM2-E and Ch-NaM2-4) are also related to the retained protons, as chitosan contents are similar to those observed for samples D, which show lower values, +1.9 and +1.3 mV for Ch-NaM2-D and Ch-NaM4-D respectively.

However, in Ch-NaM2-A and Ch-NaM4-C samples, the zeta-potential value remains negative, although the absolute vale diminishes The remaining negative value could be interpreted as the main adsorption mechanism in those conditions was thorough cation exchange reaction being the amount of outside adhered chitosan low^50,53^. Additionally, the low amount of chitosan adsorbed into the nanoclay in Ch-NaM2-A, (1.0 g/100 g, Table 1) justifies this behaviour.

**Figure 1:**
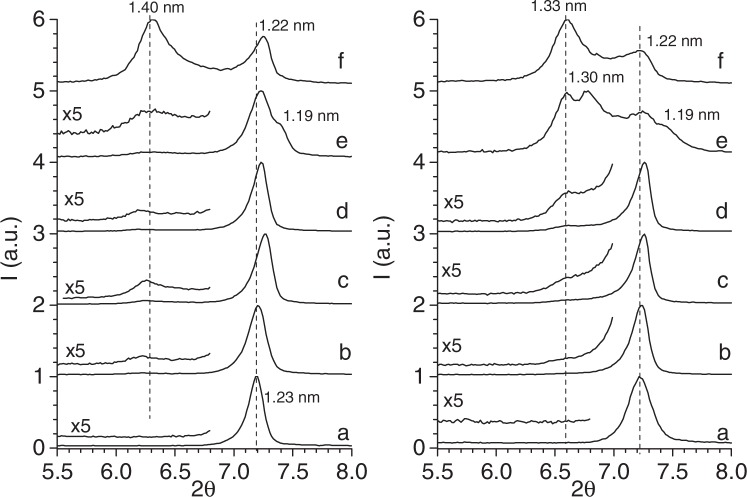
XRD in the 5–8° 2θ range of Mica-2 (*n* = 2), left graph, and, Mica-4 (*n* = 4), right graph. (**a**) NaM*n*, (**b**) Ch-NaM*n*-A, (**c**) Ch-NaM*n*-B, (**d**) Ch-NaM*n*-C, (**e**) Ch-NaM*n*-D, and, (**f**) Ch-NaM*n*-E.

X-ray diffractograms of the Na-M*n* (Figs 1a and S3a) show patterns that corresponded to that previously reported for swelling high-charged micas^54^ with a unique 001 reflection corresponding to a basal space of 1.22 nm due to hydrated Na^+^ ^55^.

The XRD patterns of mica/chitosan bionanocomposites (Fig. 1b–f) show that the reflection of the basal space of Na-M*n* does not disappear, but a new weakened broad reflection at lower angle is observed. This shift indicates the formation of an intercalated nanostructure, while the broadening and low intensity of the reflection indicates a disordered intercalated structure or a partially exfoliated structure^56^.

The increase in the basal spacing, Δd_001_ ca. 0.17 nm, is below the d_*00l*_ value measured from the XRD pattern of a chitosan film (0.38 nm)^57^ indicating that the treatment of mica with chitosan could not achieve the total intercalated state of the nanocomposite. Probably, due to the coiled structure of chitosan, intercalation only occurs in planar conformation, so that, although polymer molecules were effectively trapped inside the mica interlayer, part of them could remain at the outer surface of the clay particles^53^. Similar behaviour was observed by Günister *et al*.^50^, who concluded that chitosan molecules did not enter sufficiently into the layers of clay structures and the protons in chitosan were hydrogen-bonded to the oxygen species of Si–O and Al–O segments. In any case, there is co-existence between the original and intercalated samples.

The intensity of the low angle reflection increases with the initial chitosan concentration (Ch-NaM*n*-C vs Ch-NaM*n*-D, Fig. 1d,e, respectively), being remarkable in micas dispersed in acetic acid instead of water, Ch-NaM*n*-E (Fig. 1f). The d-spacing for the unmodified clay minerals swells when acetic acid 1% v/v is added to the aqueous dispersions^50,58^, and thus, the incorporation of chitosan into the interlayer space is facilitated thorough electrostatic interaction, compatible with the quite high positive zeta-potential in those samples.

The 2Ѳ region of the XRD patterns where the chitosan reflections appear is shown in Fig. S3. The XRD pattern of pure chitosan (Fig. S3g) is characterized by two main reflections at 9.92° and 20.19° 2Ѳ due to 002 and 200 planes^57^. The XRD pattern of the bionanocomposites, Fig. S3b-3f, show only the reflection of the starting Na-M*n*, Fig. S3a, whereas the reflections of pure chitosan, Fig. S3g, are not detected. Therefore, in all of the Ch-NaM*n* bionanocomposites, the chitosan is interacting with the mica structure as part of the bionanocomposite.

**Figure 2:**
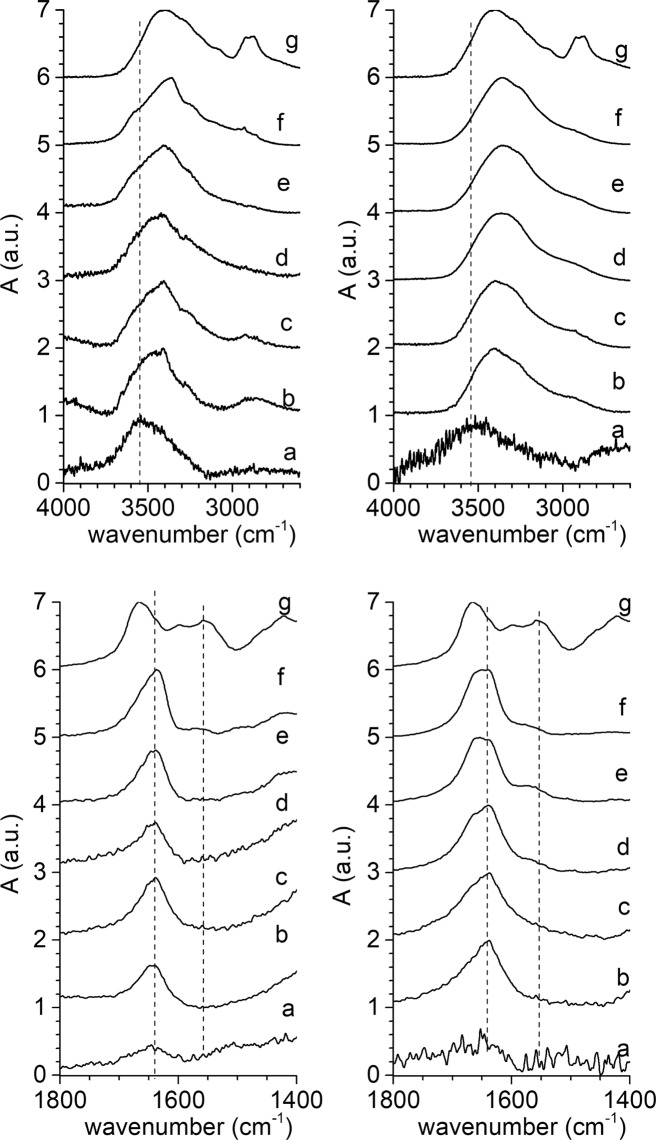
4000–2600 cm^−1^ and 1800–1400 cm^−1^ regions of the FTIR spectra of Mica-2 (*n* = 2), left graph, and, Mica-4 (*n* = 4), right graph. (**a**) NaM*n*, (**b**) Ch-NaM*n*-A, (**c**) Ch-NaMn-B, (**d**) Ch-NaM*n*-C, (**e**) Ch-NaM*n*-D, (**f**) Ch-NaM*n*-E, and, (**g**) chitosan.

A deep insight into the chitosan-mica interaction can be performed thorough the analysis of the 4000–2600 cm^−1^ and 1800–1400 cm^−1^ regions of the FTIR spectra (Fig. 2). Chitosan spectrum (Fig. 2g) shows bands at ca. 3450 cm^−1^ (interlayer O-H stretching), 3370 cm^−1^ (N-H stretching) and those close to 2900 cm^−1^ which corresponds to aliphatic C-H stretching vibrations. Additionally, the amide II band, C-O stretching of the acetyl group and N-H deformation vibration appear at 1650 and 1590 cm^−1^, respectively. Finally, the bands at ca. 1400 cm^−1^ is attributed to C-H bending^58,59^. The Na-M*n* spectra (Fig. 2a) only show a very weak band at ca. 3622 cm^−1^ (structural O-H stretching).

The infrared spectra of the chitosan-mica composites (Fig. 2-f) are formed by the combination of the mica and chitosan pure phase bands. On the one hand, in the 4000–2600 cm^−1^ region of the bionanocomposites (Fig. 2b–f, upper), the weak band at ca. 3622 cm^−1^ mica structural O-H stretching almost disappears and the N-H stretching band of chitosan emerges. On the other hand, the bands of amides and the N-H bending vibration shift to low frequency in the bionanocomposite FTIR spectra (Fig. 2b–f, bottom). As previously reported, the shifts of ν(NH_3_^+^) band confirms the interaction mainly of amino groups with mica^58,59^. Moreover, the interaction between the mica and the polymer modified the intensity of the band at 1550 cm^−1^ attributed to NH_3_^+^ which is the responsible of the intercalation by cation exchange mechanism^56^.

**Figure 3:**
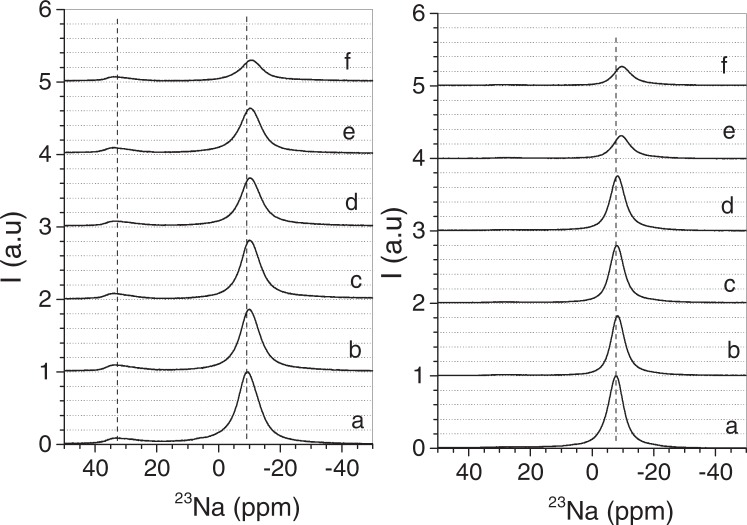
^23^Na MAS NMR spectra of Mica-2 (n = 2), left graph, and, Mica-4 (n = 4), right graph. (**a**) NaM*n*, (**b**) Ch-NaM*n*-A, (**c**) Ch-NaM*n*-B, (**d**) Ch-NaM*n*-C, (**e**) Ch-NaM*n*-D, and, (**f**) Ch-NaM*n*-E.

Although, the FTIR data are compatible with interaction chitosan/mica, the changes in the coordination sphere of the remaining interlayer sodium can only be analysed by ^23^Na MAS-NMR spectroscopy (Fig. 3). The spectra of sample Na-M*n* are characterized by two set of signals: (i) a main one in the range between 0 to −25 ppm due to hydrated interlayer sodium^60^; and, (ii) a broad signal at ca. 27 ppm from non-exchangeable sodium^61^. The ^23^Na MAS-NMR spectra of bionanocomposites indicate that the initial Na^+^ content in the mica is strongly reduced after treatment with the chitosan solutions. Even at chitosan-mica ratios < 1, the intensity of ^23^Na signal decreases. In this way, cation exchange mechanism drives the intercalation of the biopolymer into the mica substrate^20^.

**Figure 4:**
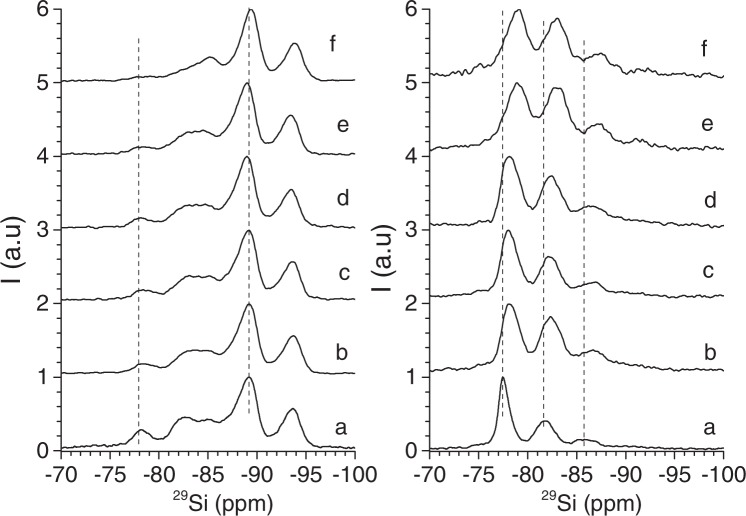
^29^Si MAS NMR spectra of Mica-2 (*n* = 2), left graph, and, Mica-4 (*n* = 4), right graph. (**a**) NaM*n*, (**b**) Ch-NaM*n*-A, (**c**) Ch-NaM*n*-B, (**d**) Ch-NaM*n*-C, (**e**) Ch-NaM*n*-D, and, (**f**) Ch-NaM*n*-E.

A deep understanding of the effect of chitosan on the mica framework is analysed by ^29^Si MAS-NMR spectroscopy. The ^29^Si MAS-NMR spectra of Na-M*n* (Fig. 4a) consist on a set of signals on the −70 to −95 ppm region due to Q^3^(mAl) (3 ≤ m ≤ 0) mica environments^40^ and in Na-M2, Q^4^(4Al) from sodalite^62^. Due to the different number of isomorphous substitutions in the mica tetrahedral sheet, a shift is observed in all the Q^3^(mAl) signals in NaM2 sample, accompanied by a different relative intensity ratios between them. The relative intensity of each ^29^Si Q^3^(mAl) signals are the same as previously reported for Na-M*n*^40^.

The ^29^Si MAS-NMR signals of Ch-NaM2 remain at the same chemical shifts but those of the Ch-NaM4 (Fig. 4b–f) shift to lower frequency, being more evident as the amount of chitosan in the bionanocomposite increases (Table 1). Alba *et al*.^46^ observed the same effect in the ^29^Si MAS-NMR signals signals of O-Mica-*n* (O is alkylammonium cation), justifying it with the incorporation of the NH_3_^+^ group into the hexagonal hole formed by the Si/Al tetrahedra, the optimal tilt angle being 50–51° ^63^.

However, XRD results showed that chitosan molecules are intercalated in a planar conformation, with part of them outer attracted to the mica particles^53^. Chitosan protons are, hence, hydrogen-bond to the Si-O and Al-O bonds in the tetrahedral sheet, provoking a weakness in those bonds and a distortion in the tetrahedral symmetry of these moieties, and, consequently, a shift in the ^29^Si signals^50^.

Additionally, the relative intensity of the Q^3^(mAl) signals of the Ch-NaM4 change respect to the pure Na-M4. An increasing enrichment of Q^3^(mAl), m ≤ 2, environments is observed that can be caused by a decrease of the total layer charge and could also contribute to the lower frequency shift of the signals^40^. Alba *et al*.^64^ found that in acid media the tetrahedral aluminium can leach generating an increase in the Si/Al ratio and, consequently, an intensity decreases in the Q^3^(mAl) sites rich in aluminium increases and Q^3^ rich in aluminium decreases.

In the case of Ch-NaM2, the relative intensity of the signals remains almost constant except for Ch-NaM2-E, mica dispersion with acetic acid, (Fig. 4) where the Q^3^(3Al) environment almost disappears. That shows that the intercalation of chitosan in the low charge nanoclay does not perturb the mica framework.

## Summary and Conclusions

For the first time, bionanocomposites based on the combination of chitosan with designed swelling high-charged micas have been prepared.

In general, the bionanocomposite prepared from NaM4 contains more chitosan than those from NaM2.Those bionanocomposites exhibits higher thermal stability than the pure chitosan.

The XRD, FTIR, ^29^Si and ^23^Na MAS-NMR spectroscopy results indicate that chitosan is incorporated into the mica but part of them remains at the outer surface being hydrogen-bonded to the Si-O and Al-O of the basal plane. The surface coverage is quite high and the zeta-potentials change from a negative value of Na-M*n* to a positive value for the bionanocomposites.

The most favourable chitosan loading condition was attained with a chitosan/mica ratio of 5 wt. and a 5 mg/ml solution of chitosan in acetic acid at 1%. The dispersion medium of mica has a minor influence, although optimum conditions is obtained in acetic medium.

## Methods

### Synthesis of Mica

The synthesis method employed was that described by Alba *et al*.^40^. Powder mixtures with molar composition: (8 - *n*) SiO_2_, (*n*/2) Al_2_O_3_, 6 MgF_2_, and (2*n*) NaCl (*n* is the layer charge per unit cell, *n* = 2 and 4) were used. The starting materials were SiO_2_ from Sigma (CAS no. 112945-52-5, 99.8% purity), Al(OH)_3_ from Sigma Aldrich (CAS no. 21645-51-2, 99% purity), MgF_2_ from Aldrich (CAS no. 20831-0, 98% purity), and NaCl from Panreac (CAS no. 131659, 99.5% purity). All reagents were mixed and ground in an agate mortar and, then, they were heated in air up to 900 °C for 15 h, in a Pt crucible. Finally, the solids were washed with deionized water and dried at room temperature. The as-synthesized samples were named as Na-M*n* (*n* = 2 and 4).

### Synthesis of bionanocomposites

Chitosan (Ch) with 310000-375000 Da of molecular weight and a viscosity of 1210 CPS was supplied by Aldrich (Product number 419419). A molecular structure of the chitosan has been included in Fig. S1.

Chitosan solutions were prepared by the addition of the corresponding amounts of polysaccharide to 10% (v/v) or 1% (v/v) acetic acid (see Table 2) proportion chitosan: acetic solution). The solution was stirred for 4 h and the pH adjusted to 4.9 with NaOH solution before mixing with the clay dispersion. Chitosan solutions containing variable weight of biopolymer were slowly added to a 2% mica dispersion (0.5 g of Na-M*n* in 25 mL of bidistilled water or 1% (v/v) acetic acid) and stirred for 2 h, at 323 K, to obtain the bionanocomposites, Ch-NaM*n*-X (*n* = 2 or 4, and X = A, B, C, D or E, see Table 2).

**Table 2:** Synthesis parameters and sample name of Ch-NaM*n* (*n*=2 and 4).

*n*	Ch:Mica	Ch solution			Mica	sample
	wt	Acetic acid	Ch:acid (mg/ml)	pH after 4 h	solvent	
2	0.42	10%	1.70	2.2	water	Ch-NaM2-A
4	0.80	10%	3.22	2.3	water	Ch-NaM4-A
2	0.42	1%	1.70	3.2	water	Ch-NaM2-B
4	0.80	1%	3.22	3.4	water	Ch-NaM4-B
2	2	1%	4	3.6	water	Ch-NaM2-C
2	5	1%	5	3.6	water	Ch-NaM2-D
4	2	1%	4	3.6	water	Ch-NaM4-C
4	5	1%	5	3.6	water	Ch-NaM4-D
2	5	1%	5	3.5	Act.1%	Ch-NaM2-E
4	5	1%	5	3.5	Act.1%	Ch-NaM4-E

The resulting mixture was stirred for 48 h and washed with purified water. The nanocomposites were air-dried and ground to powder.

### Characterization

Thermogravimetric analysis, TG, were carried out using a TA (model STD-Q600) instrument, in the Characterization Service (CITIUS, University of Seville, Spain), with alumina as reference. The samples were placed into a Pt crucible and maintained in air throughout the heating period. The temperature was increased at a constant rate of 10 °C/min. The temperature of each loss weight on the TG was determined thorough the derivative TG curve as a function of temperature (DTG).

Electrokinetic potentials were determined in a Malvern Zetasizer nano-zs90 in the Colloidal Materials Laboratory of ICMS. For each determination, 2.5 mg of samples were dispersed in 5 mL of a 10^−3^ M KCl solution, the slurry was stirred and pH was measured, the natural pH value for all the bionanocomposites was ca. 6 and for Na-M2 and Na-M4 that was ca. 9 and, thus, it was adjusted to 6 by adding 0.1 M HCl solution.

X-ray diffraction (XRD) patterns were obtained at the X-ray laboratory (CITIUS, University of Seville, Spain) on a Bruker D8 Advance instrument equipped with a Cu K_α_ radiation source operating at 40 kV and 40 mA. Diffractograms were obtained in the 2θ range of 1.5–70° with a step size of 0.015° and a step time of 0.1 s.

FTIR spectra were recorded in the range 4000–300 cm^–1^ in the Spectroscopy Service of the ICMS (CSIC-US, Seville, Spain), as KBr pellets dried at 120 °C, using JASCO FT/IR-6200 IRT-5000 instrument.

Single-pulse (SP) MAS-NMR experiments were recorded on a Bruker AVANCE WB400 spectrometer equipped with a multinuclear probe, in the Nuclear Magnetic Resonance Service of University of Córdoba (Córdoba, Spain). Powdered samples were packed in 3.2 mm zirconia rotors and spun at 10 kHz. ^29^Si MAS-NMR spectra were acquired at a frequency of 79.49 MHz, pulse width of 2.7 µs (π/6) each 3 s. ^23^Na MAS-NMR spectra were recorded at 105.84 MHz with a pulse of 0.75 µs (π/12) and a delay time of 0.1 s. The chemical shift values were reported in ppm from tetramethylsilane for ^29^Si and from a 0.1 M NaCl solution for ^23^Na.

## Supplementary information

Supplementary material
