# Supplementary material for: Bionanocomposites based on chitosan intercalation in designed swelling high-charged micas
Source: Sci Rep. 2019 Jul 16;9:10265. doi: 10.1038/s41598-019-46495-z (PMC6635363; doi:10.1038/s41598-019-46495-z)
Supplement: Supplementary file 1 — Supplementary material [file 41598_2019_46495_MOESM1_ESM.docx]

**Bionanocomposites based on chitosan intercalation in designed swelling high-charged micas**

María D. Alba^[[1]](#footnote-1)^, Agustín Cota^[[2]](#footnote-2)^, Francisco J. Osuna^1^, Esperanza Pavón^1^, Ana C. Perdigón^[[3]](#footnote-3)^ & Florian Raffin^[[4]](#footnote-4)^.

| **Parameters** | **values** | **nomenclature** | |
| --- | --- | --- | --- |
|  |  | **Na-Mica2** | **Na-Mica4** |
| Acetic acid  concentration | 10% | Ch-NaM2-A | Ch-NaM4-A |
|  | 1% | Ch-NaM2-B | Ch-NaM4-B |
| Ch:mica (wt) | 0.42 | Ch-NaM2-B | -- |
|  | 0.80 | -- | Ch-NaM4-B |
|  | 2 | Ch-NaM2-C | Ch-NaM4-C |
|  | 5 | Ch-NaM2-D | Ch-NaM4-D |
| Solvent of mica  suspension | Water | Ch-NaM2-D | Ch-NaM4-D |
|  | Acetic acid | Ch-NaM2-E | Ch-NaM4-E |

**Table S1.** Summary of the evaluated synthesis parameters and nomenclature of the samples.

**Figure S1.** Chitosan structure. N: blue; O: red; C: dark grey; and; H: light grey.

**Figure S2.** TG (red dash line) and DTG (black solid line) plots in the adsorbed water loss region (25 ºC-200 ºC) and chitosan decomposition region (200 ºC-900 ºC) of Mica-2 (n=2), left graph, and, Mica-4 (n=4), right graph. a) Ch-NaMn-A, b) Ch-NaMn-B, c) Ch-NaMn-C, d) Ch-NaMn-D, e) Ch-NaMn-E, and, f) chitosan.

**Figure S3.** XRD pattern in the 2Ѳ range between 8º and 40 º of Mica-2 (*n*=2), left graph, and, Mica-4 (*n*=4), right graph. a) NaM*n*, b) Ch-NaM*n*-A, c) Ch-NaM*n*-B, d) Ch-NaM*n*-C, e) Ch-NaM*n*-D, f) Ch-NaM*n*-E, and, g) chitosan

1. Instituto Ciencia de los Materiales de Sevilla, ICMS, (CSIC-US), Avda. Americo Vespucio, 49, 41092 Sevilla, Spain (alba@icmse.csic.es). [↑](#footnote-ref-1)
2. Laboratorio de Rayos X, CITIUS, (Universidad de Sevilla), Avda. Reina Mercedes, 4, 41012 Sevilla, Spain [↑](#footnote-ref-2)
3. Departamento de Química e Ingeniería de Procesos y Recursos. Universidad Cantabria. Avda. Los Castros s/n. 39005 Santander, Spain. [↑](#footnote-ref-3)
4. École Nationale Supérieure de Chimie de Lille (E.N.S.C.L). Cité Scientifique – Bât 7. Avenue Mendeleïev CS 90108. 59652 Villeneuve D’ascq Cedex, France.

   Correspondence and requests for materials should be addressed to Dr. M.D. Alba (alba@icmse.csic.es) [↑](#footnote-ref-4)
